# Mid-term results show no significant difference in postoperative clinical outcome, pain and range of motion between a well-established total knee arthroplasty design and its successor: a prospective, randomized, controlled trial

**DOI:** 10.1007/s00167-020-06027-z

**Published:** 2020-04-24

**Authors:** Georg Hauer, Nina Hörlesberger, Sebastian Klim, Gerwin A. Bernhardt, Lukas Leitner, Mathias Glehr, Andreas Leithner, Patrick Sadoghi

**Affiliations:** Department of Orthopaedics and Trauma, Medical University of Graz, Auenbruggerplatz 5, 8036 Graz, Austria

**Keywords:** Total knee arthroplasty, Total knee replacement, TKA, Outcomes, Attune, PFC sigma

## Abstract

**Purpose:**

The purpose of this study was to compare the clinical and functional outcome scores following total knee arthroplasty (TKA) with two different systems. The hypothesis was that there is a difference between patients receiving the newer design than those receiving the predecessor.

**Methods:**

Two hundred patients who underwent TKA were randomized into two groups: patients received either Attune TKA or PFC Sigma (both DePuy Synthes, Warsaw, IN). Clinically, the Knee Society Knee and Function Scores (KS and FS), Western Ontario and McMaster Universities Osteoarthritis Index (WOMAC), Range of Motion (ROM) and Visual Analogue Scale (VAS) were evaluated and compared between the groups 2 years after surgery. 158 patients (80 in the Attune group and 78 in the PFC Sigma group) were available for follow-up.

**Results:**

Through bivariate analysis using parametric and non-parametric statistical tests, no significant differences in postoperative KS, FS, WOMAC, ROM or VAS between the two groups were detected. Both groups significantly improved regarding all evaluated endpoints 2 years after surgery.

**Conclusions:**

In the current study population, no difference in clinical outcome between the two systems was found. The expected benefits of design modifications could not be observed in clinical outcome scores 2 years postoperatively. Both designs are effective options for improving pain and function in end-stage osteoarthritis.

**Level of evidence:**

I.

## Introduction

Although TKA is a highly effective procedure, up to 20% of patients continue to suffer from pain after the replacement of the joint [5]. To address this difficulty, the number of implants available on the market has substantially increased, often with little or no evidence of clinical effectiveness or cost-effectiveness [5].

According to the national arthroplasty registry data, the Press Fit Condylar (PFC) Sigma (DePuy Synthes, Warsaw, IN) was one of the ten most used prostheses in primary TKA [1, 9] worldwide. Despite very promising survival rates after long-term follow up, the PFC Sigma has continued to be associated with relatively high rates of dissatisfaction at short-to-midterm follow-ups [10]. In 2013, the company revealed a modification of the design. The Attune TKA System (DePuy Synthes, Warsaw, IN) was designed to address these rates by optimising the guidance in femoral size measurement and the flexion gap balancing using a single tool [11]. The new prosthesis was developed to more closely resemble the anatomical trochlear groove and patella as patellofemoral maltracking, tilt, and overstuffing have been shown to be important factors leading to pain and thus to unsatisfying results [4].

As the two TKA designs are amongst the most frequently used knee replacement systems worldwide, their clinical outcome is of great importance for orthopaedic surgeons [1, 9]. The current literature suggests that both implants perform equally well, but well-designed, independent studies on the subject are required to clearly evaluate the effect of the implant design on the postoperative functional outcome [6, 8, 10, 12]. This paper gives further insight into this evaluation as no prospective, randomized, controlled trial has yet been conducted to compare both designs. Therefore, the aim of this study was to compare the well-established PFC system and the Attune TKA using clinical outcome scores after 2-year follow-ups. The study’s hypothesis was that modifications of the design of the Attune system would lead to an improved clinical outcome compared to the PFC Sigma system.

## Methods

A prospective, randomized, parallel-group study was conducted in a single, urban, high-volume university hospital. Participants were recruited from the orthopaedic outpatient clinic. During their attendance patients were assessed both for their eligibility and their willingness to participate. Patients were eligible for inclusion in the trial if a decision had been made to have primary total knee arthroplasty surgery, but no particular type of prosthesis had been determined. All the patients were suffering from severe osteoarthritis of the knee confirmed by anteroposterior and lateral radiographs (Kellgren-Lawrence-Score III/IV) and high levels of pain in at least two knee compartments despite conservative treatment. Exclusion criteria were a flexion of less than 70° (*n* = 7), varus and valgus deformities of more than 15° (*n* = 11), patients with known or suspected metal allergies (*n* = 4) and patients under 60 years of age (*n* = 35). Patients who were unwilling to participate (*n* = 2) or unable to provide informed consent (*n* = 1) were also excluded.

Included patients were randomized and received either an Attune or a PFC Sigma TKA (both DePuy Synthes, Warsaw, IN). Participants were randomly assigned 1:1 the day before surgery using the Randomizer for Clinical Trials tool developed at the Medical University of Graz. A study nurse not involved in participant recruitment and patient care prepared the randomization in advance. Neither the patients nor the surgeons were blinded to the treatment allocation. The recruitment of study participants finished as soon as 100 patients were included in each group.

All procedures were carried-out by two senior knee surgeons using the same surgical technique with no patella resurfacing. Femoral and tibial components were cemented (Palacos R + G, Heraeus Medical, Wehrheim, Germany) and the tibia first method with the balancing of the flexion gap was carried out using a medial parapatellar approach. Postoperatively, patients followed a standardized rehabilitation protocol consisting of full weight bearing immediately after surgery with the use of crutches and continuous passive motion (CPM) therapy on the first postoperative day.

### Study population

**Fig. 1:**
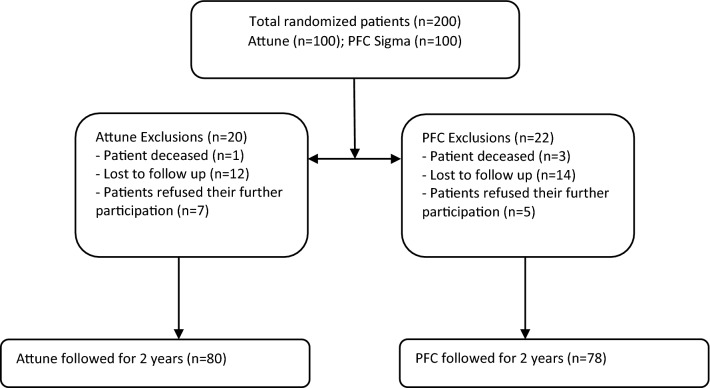
Flow chart on study inclusion

Out of two hundred consecutively enrolled patients, 80 patients in the Attune group and 78 patients in the PFC group were assessed at the 2-year follow-up analysis. A patient flow chart on study inclusion has been provided in Fig. 1. One patient in the Attune group and three patients in the PFC group had died of causes unrelated to their knee surgery. Twelve patients refused their further participation in the study at a final follow-up examination. Twenty-eight patients were lost to follow-up as they could not be contacted or did not appear on the scheduled examination dates. According to a systematic search of the patients’ medical records, none of the patients lost to follow up or who refused their further participation had undergone revision surgery in the center hospital or regional partner hospitals. There were no significant differences in the demographic data between the two groups (Table 1).

**Table 1:** Patient demographics and baseline characteristics show no significant (n.s.) differences between our study groups

Demographics	Attune (*n* = 80)	PFC (*n* = 78)	*p* value
Sex (M/F) (*n*/%)	28(35%)/52(65%)	28 (35.9%)/50(64.1%)	n.s
Age (years), mean (SD)	71.1 (8.3)	69.0 (8.0)	n.s
BMI (kg/m^2^), mean (SD)	28.8 (4.4)	29.4 (4.6)	n.s

### Outcome measurement

The preoperative clinical status and results 2 year postoperatively were evaluated according to Knee Society Knee and Function Scores (KS and FS) [7], the Western Ontario and McMaster Universities Osteoarthritis Index (WOMAC) [3], the Visual Analogue Scale (VAS), and range of motion (ROM). The ROM was measured with a double-armed goniometer.

The study procedure followed accepted ethical, scientific, and medical standards and was conducted in compliance with recognized international standards, including the principles of the Declaration of Helsinki. Informed consent was obtained from all the study’s participants and the study protocol was approved by the ethics committee of the Medical University of Graz (26-303 ex 13/14).

### Statistical analysis

The data were analysed by SPSS Version 23.0 (IBM Corporation, New York, USA). Descriptive statistics for continuous variables were reported as the mean and standard deviation (SD). Categorical variables were reported as count and proportions. For comparisons of categorical variables, the chi-square exact test was used. The data were tested for normality using the Kolmogorov–Smirnov test, which revealed a parametric distribution for the BMI and a non-parametric distribution for all clinical scores and ROM. Differences between pre-operative and post-operative data were observed through the *t *test and the Mann–Whitney *U* test and the Wilcoxon signed-rank test. A *p* value of < 0.05 was interpreted as being statistically significant. An a priori power analysis revealed that a minimum of 64 patients per group would be necessary to detect a clinically relevant difference of 10° in ROM with a SD of 20° at a significance level of *p* < 0.05 with a power of 0.8. To compensate for dropouts, 100 study participants per group was considered a sufficient number.

## Results

### Clinical results

There were no significant differences in terms of pre- and postoperative KS and FS, WOMAC, VAS and ROM between the two groups (Table 2). At the 2-year follow-ups, statistically significant (*p* < 0.001) improvements were obtained in all clinical scores and ROM within the groups compared to preoperative values (Table 2). In the final follow-up cohorts, no minor or major complications that required further surgical treatment were observed.

**Table 2:** Comparison of Clinical Outcome before and after 2-year follow-up

	Attune (*n* = 80)	PFC (*n* = 78)	*p *value
ROM (°) (mean ± SD)			
Pre-operative	93.4 ± 21.8	95.9 ± 17.7	n.s
Post-operative	113.0 ± 10.6	112.3 ± 11.5	n.s
Change in ROM	21.8 ± 19.8	21.5 ± 15.6	n.s
WOMAC (mean ± SD)			
Pre-operative	53.7 ± 14.0	55.6 ± 15.2	n.s
Post-operative	86.7 ± 15.3	88.5 ± 12.9	n.s
Change in WOMAC	21.6 ± 19.7	18.1 ± 12.0	n.s
KS (mean ± SD)			
Pre-operative	51.5 ± 14.6	52.3 ± 13.6	n.s
Post-operative	92.6 ± 10.4	88.8 ± 14.3	n.s
Change in KSS pain	42.0 ± 16.8	36.6 ± 16.6	n.s
FS (mean ± SD)			
Pre-operative	44.6 ± 12.2	44.3 ± 18.4	n.s
Post-operative	85.0 ± 19.3	79.8 ± 21.8	n.s
Change in KSS function	41.8 ± 21.7	38.9 ± 20.9	n.s
VAS (mean ± SD)			
Pre-operative	6.6 ± 2.1	6.5 ± 1.9	n.s
Post-operative	1.6 ± 1.5	1.6 ± 1.8	n.s
Change in KSS function	5.1 ± 2.3	5.0 ± 2.1	n.s

Clinically and statistically, there were no significant (n.s.) differences in terms of pre- and postoperative ROM, WOMAC, KS, FS and VAS

## Discussion

The most important finding of this study was that both systems provided excellent clinical results at the 2-year follow-ups. However, no superiority of the Attune TKA concerning clinical outcome scores and ROM could be detected. This study adds to the literature as it represents the first, randomized, controlled trial to compare these two systems.

Studies have already been carried out to provide data following Attune and PFC TKA and this current study is in line with previous reports [6, 8, 10, 12]. The new anatomical design was established to improve function and reduce knee pain, however, this could not yet be clearly observed. Ranawat et al. [10] found that in a 2-year follow-up matched-pair analysis no significant differences between the two groups in KS pain or function scores could be observed. The satisfaction rate for the Attune group was 97.9% and for the PFC group 96.6%. According to the authors, modifications to the patellofemoral joint did not result in significant improvements. Song et al. [12] showed more satisfactory results with the Attune TKA than the PFC regarding the KS, FS, WOMAC, and ROM after 2 years. In this study, a trend towards more favorable clinical results in the Attune group was detected with respect to ROM, KS and FS, however, without any statistical significance. Similar findings were also reported by Chua et al. [6], who reported that the newer TKA and the predecessor design achieved comparable improvements after 2 years. These authors even question the legitimacy of using a newer and costlier design in the absence of clear benefits. Another retrospective analysis by Molloy et al. [8] demonstrated that Attune implants do not appear to improve ROM and physical function outcomes relative to PFC.

The primary goal of the modification of a well-established implant should be an improvement of functional outcome and a decrease of unsatisfied patients. However, according to the present findings and aforementioned publications, theoretical advantages of the new design did not result in improvement of clinical outcome scores. It seems that the design might not be the sole key factor when it comes to functional outcome scores [2]. Other factors like the surgeon, the implantation technique, the surgical approach, the pain management and the patient characteristics might be just as important when determining the success of an implant. Behrend et al. [2] state that possibly only custom-made implants can reproduce the anatomy sufficiently and that off-the-shelf prostheses might not offer room for further improvement [2]. Therefore, considering the usually higher costs of new developments, clinical and functional outcome scores are important indicators to prove economic reasonability before establishing and justifying the routine use of newer designs [6].

According to the local hospital database, all of the initial patients are free of revision surgery. However, it has recently been published that Attune prostheses show highly significant differences in the occurrence of radiolucent lines compared to PFC Sigma [13]. It will remain interesting if this occurrence leads to increased rates of aseptic loosening. Nonetheless, to date, there are no significant differences in terms of revision rates between the two prostheses in arthroplasty registers [1, 9].

There are several limitations to the study. First, we had a relatively high dropout rate, which could represent a selection bias. Although our study nurse contacted patients several times, most of them stated, that they were satisfied and therefore not willing to participate in the follow-up evaluation. Second, although we used validated clinical scores to evaluate postoperative pain and function, these clinical rating systems may not be sensitive enough to detect subtle changes. Therefore, a ceiling effect must be considered when interpreting the findings of this study. Third, our surgeons have already had a lot experience with the PFC Sigma, whereas especially in the initial study phase, the Attune TKA was relatively new. A higher skill level with respect to the PFC Sigma might have confounded the postoperative outcome. Despite these limitations, this first prospective, randomized, controlled study provides additional and valuable information on the comparison between a classic implant and its evolutional successor. Furthermore, the results emphasize the need for a prolonged follow-up evaluation.

## Conclusion

The expected benefits of the Attune TKA modifications compared to its predecessor could not be observed in clinical outcome scores 2 year postoperatively. However, this study objectively demonstrated that both designs are effective options for improving pain and function following TKA. The results underscore the importance of qualitative research to clarify the performance of a traditional implant and its evolutional design. This information especially applies to surgeons when planning to change from a well-functioning implant, in which all characteristics and associated pitfalls are known, to a new, unknown design with missing practical experience.
